# Data on fluoride concentration levels in semi-arid region of Medak, Telangana, South India

**DOI:** 10.1016/j.dib.2017.11.089

**Published:** 2017-12-06

**Authors:** Adimalla Narsimha, Venkatayogi Sudarshan

**Affiliations:** Department of Applied Geochemistry, University College of Science, Osmania University, Hyderabad 500007, India

## Abstract

According to the World Health Organization recommendation, the optimal fluoride concentration levels in drinking water have to be in the range of 0.5 and 1.5 mg/L since this permissible range is essential for normal mineralization of bones and teeth as well as for dental enamel formation in human's body Bell and budwig, 1970;Adimalla and Venkatayogi, 2017;Narsimha and Sudarshan, 2013,2016;^2017^[1], [2], [4], [5], [6]. If continues intake of high fluoride (>1.5) water can severely cause dental and skeletal fluorosis. The investigated area people majorly depend on groundwater for drinking purposes and fluoride concentration ranged from 0.2 to 7.4 mg/L with mean concentration of 2.7 mg/L and data was compared with WHO guidelines for drinking purposes. Overall, data reveals that the 57% of groundwater samples data was not safe for drinking purposes. Therefore, distribution of fluoride in the groundwater of Medak region in Telangana was suggested to intake drinking water, which are below level of fluoride concentration in the groundwater and take care about health implications.

**Specifications Table**

**Table:** 

Subject area	*Earth Science*
More specific subject area	*Hydro-geochemistry*
Type of data	*Table, figure*
How data was acquired	*The fluoride concentration in groundwater was determined electrochemically, using a fluoride ion-selective electrode (ISE) with an Orion 4 star meter benchtop pH/ISE meter*[3]*. Standard fluoride solutions (0.1-10 mg/L) were prepared from a stock solution (10 mg/L) of sodium fluoride. The ion meter was calibrated for a slope of −59.2±2*[3]*.*
	*Calcium, magnesium, chloride, carbonate and bicarbonate were analyzed by a titration method.*
	*Sodium and potassium concentrations were determined using a flame photometer (Systronics, 130). Sulphate and nitrate were determined using a UV-visible spectrophotometer (Spectronic, 21, BAUSCH and LOMB).*
	*Using pH/EC/TDS meter (Hanna HI 9811-5), the EC, pH and TDS of water samples were measured.*
Data format	*Analyzed*
Experimental factors	*Brief description of any pretreatment of samples*
Experimental features	*Very brief experimental description*
*Data source location*	*Medak region, Telangana, South India.*
Data accessibility	*Data is with this article*

**Value of the Data**

• Data can be helpful as an indicator for fluoride concentration in different geological terrains (granite, basalts, and laterites) groundwater.
• The data set is very useful to hydrologist, geochemists, environmental researchers and scientists as an indicator for further take necessary steps to execute the groundwater quality research and its vulnerability estimation.
• The distribution pattern of fluoride level/concentration will act as a geochemical baseline, to delineate the groundwater quality and health implications.
• Since the dataset is geo-referenced, it can be used for geospatial modelling in GIS.

## 1 Data

**Fig. 1:**
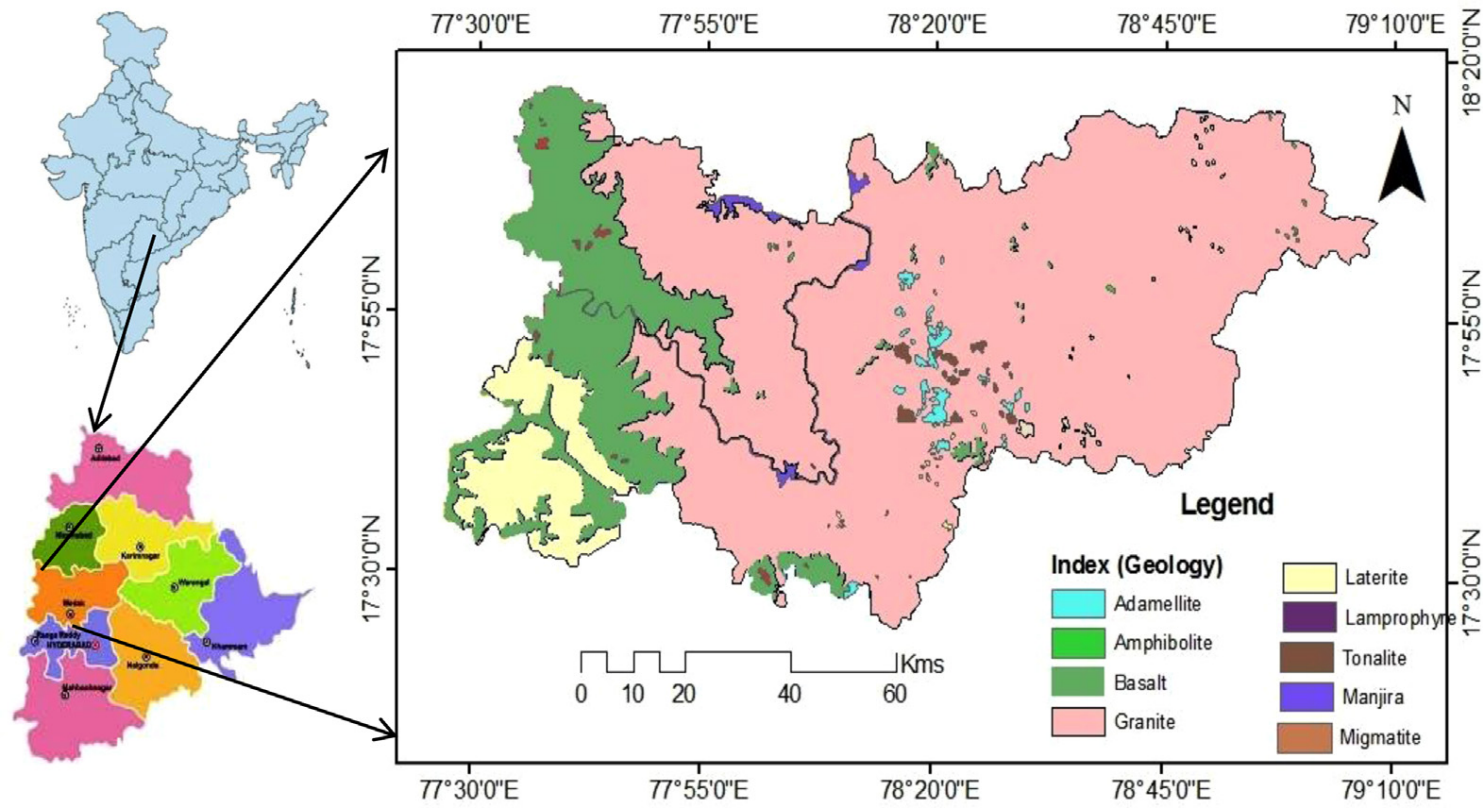
Geology map of Medak region, Telangana State, South India.

**Fig. 2:**
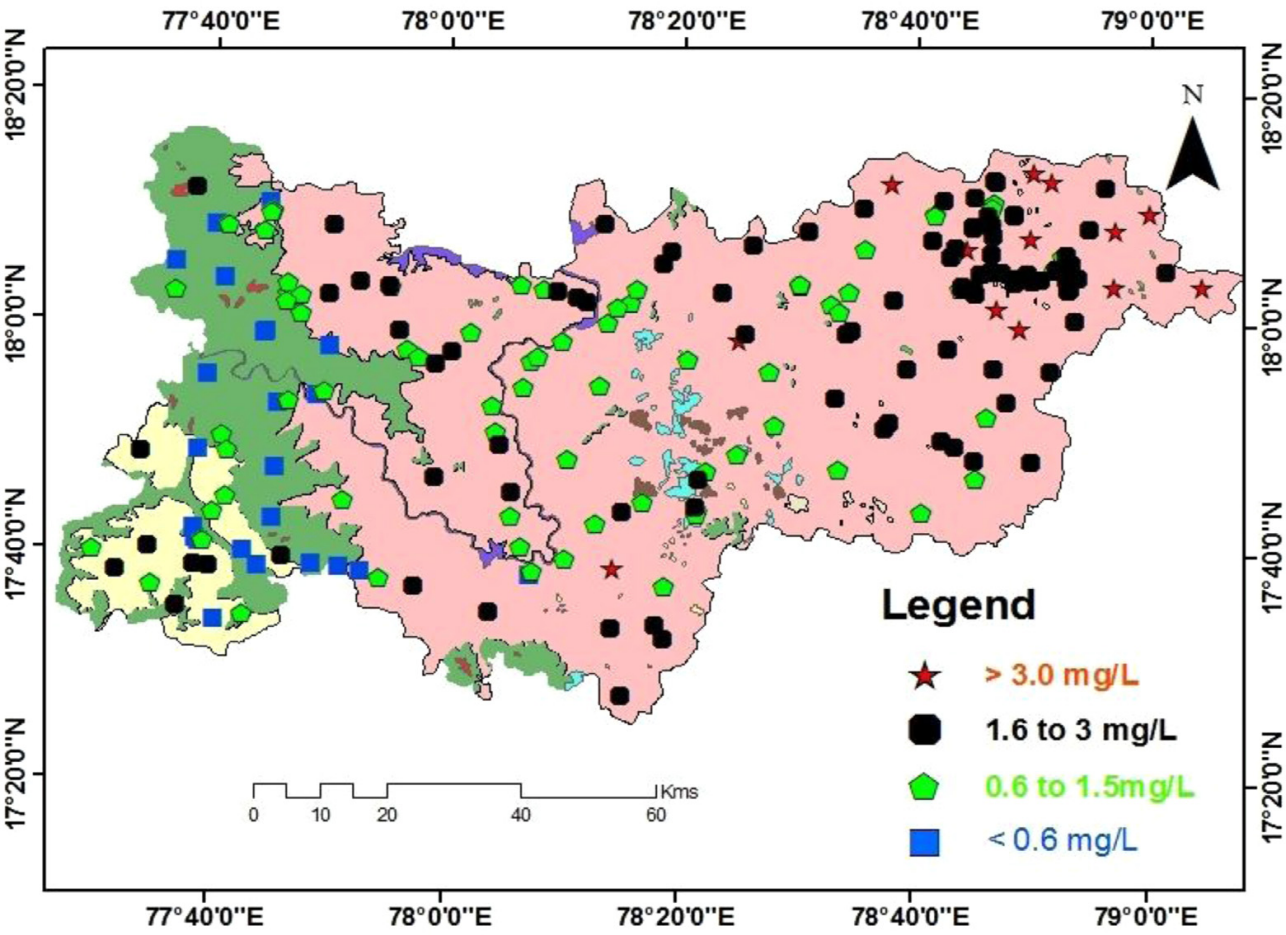
Sampling locations with different fluoride concentrations on groundwater of Medak region.

**Fig. 3:**
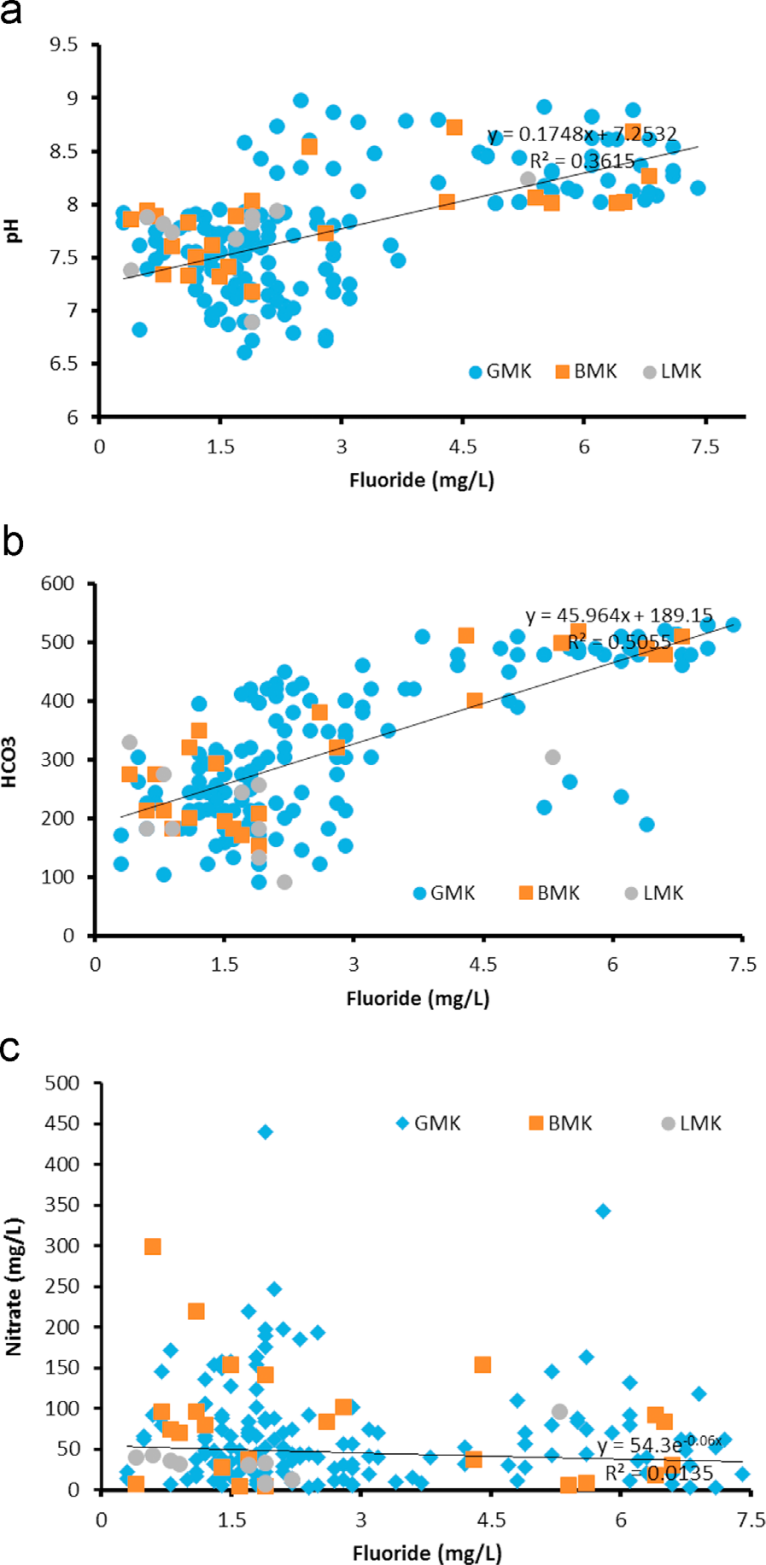

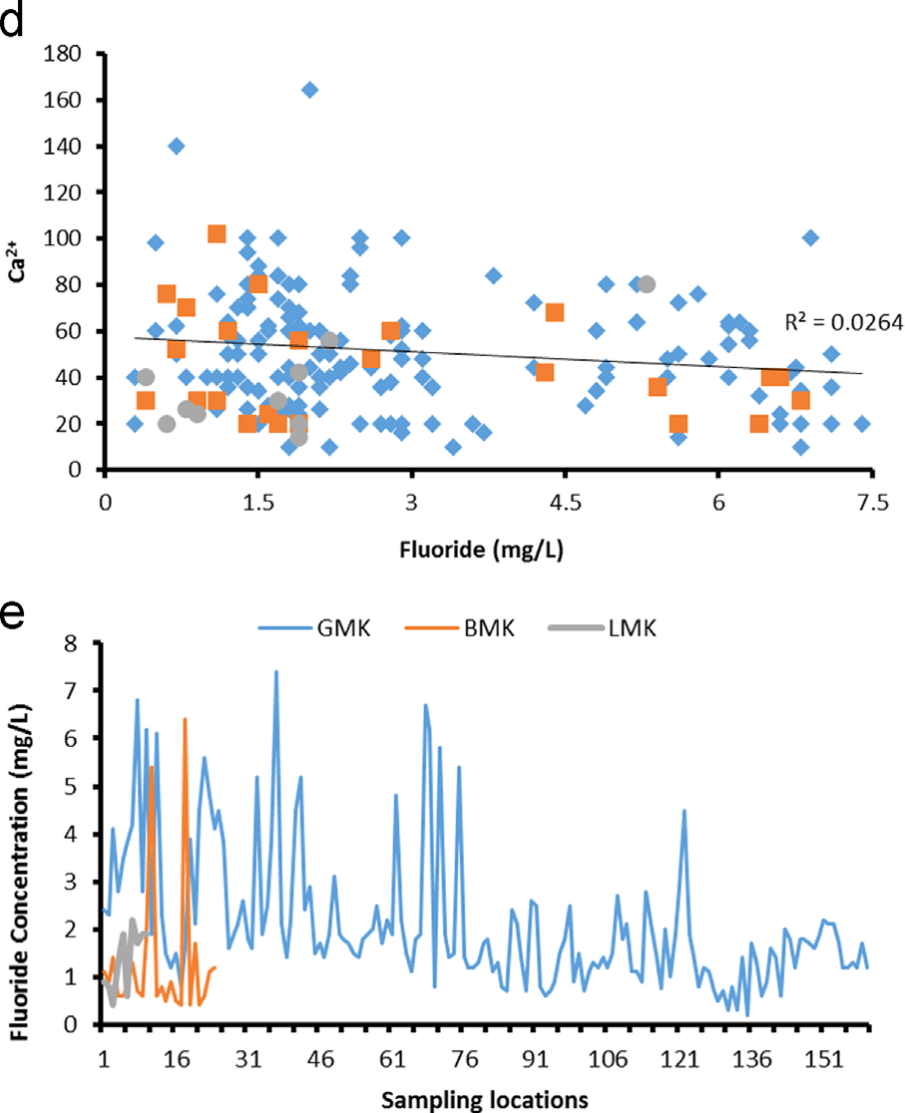
Relation between a) Fluoride and pH, b) F^-^ and ${\mathrm{HCO}}_{3}^{2-}$ c) F^-^ and ${\mathrm{NO}}_{3}^{-}$ d) F^-^ and Ca^2+^ e) fluoride concentrations and different aquifers.

Geologically, in the Medak region majorly occupied by granitic, basaltic, and laterite rocks as depicted in Fig. 1 and groundwater sampling data map as well as fluoride concentrations in groundwater data is shown in Fig. 2. Fluoride concentrations are 0.2–7.4 mg/L (GMK: Granitic area), 0.4–6.4 mg/L (BMK: Basaltic area), and 0.4 to 2.3 mg/L (LMK: laterites area), are shown in Table 1, with WHO guideline approached. pH, HCO^3-^, ${\mathrm{NO}}_{3}^{-}$, Ca^2+^ versus fluoride cross plots are shown in Fig. 3 [1], [2].

**Table 1:** Physical and chemical characteristics of groundwater samples and their comparison with WHO in different aquifers, Medak district, Telangana State, South India.

	pH	EC	TDS	TH	F^-^	${\mathrm{HCO}}_{3}^{–}$	Cl^-^	${\mathrm{SO}}_{4}^{2-}$	${\mathrm{NO}}_{3}^{-}$	Ca^2+^	Mg^2+^	Na^+^	K^+^
Minimum	a6.6	169	108	50	0.2	18	25	21	4	10	2.4	14	1
	b7.18	234	150	100	0.4	31	36	23	4	20	10	18	1
	c6.89	384	246	115	0.4	92	36	70	6.6	14	55	44	1
													
Maximum	a8.79	3170	2029	1550	7.4	527	959	328`	440	164	380	145	24
	b8.72	2350	1504	870	6.4	323	604	249	299	102	680	134	21
	c8.24	880	563	375	2.2	329	355	254	97	80	195	95	4
													
Mean	a7.56	864.9	554	240.22	2.76	306.44	245	158.4	69	52	72.0	65.36	3.2
	b7.83	794.2	508.3	240	1.51	200.5	213.13	155.12	80.45	45.67	122.35	51.25	2.83
	c7.73	583.23	373.27	212.50	1.36	218.38	146.26	168.15	33.92	35.27	124.50	67.30	1.40
													
WHO highest limits	6.5–9.2	1500	1500	500	1.5	600	600	600	45	200	150	200	12
													
% of sample exceeding highest limit	aNil	10	1	2	57	Nil	1	Nil	51	Nil	10	Nil	2
	bNil	15	1	1	17	Nil	1	Nil	58	Nil	21	Nil	4
	cNil	Nil	Nil	Nil	50	Nil	Nil	Nil	1	Nil	30	Nil	Nil

a Granitic.

b Basaltic.

c Laterite aquifers.

## 2 Experimental design, materials, and methods

Medak district is located in the central part of Telangana, South India. It lies between North Latitudes 17° 27′ and 18° 18′ and East longitude 77° 28′ and 79 °10′ falling in topographical sheet nos. 56 F, G, J and K of the Survey of India, and covers an area of 9,699 Sq. Km (Fig. 1).

One hundred and ninety four groundwater samples were collected, from bore wells/hand pumps in November 2015 in pre-cleaned 1 L polyethylene bottles [3]. The groundwater samples were analyzed for hydro-chemical parameters including pH, electrical conductivity (EC), total hardness (TH) as CaCO_3_, total dissolved solids (TDS), calcium (Ca^2+^), magnesium (Mg^2+^), sodium (Na^+^), potassium (K^+^), chloride (Cl^-^), sulphate $\left(\mathrm{SO}4^{2-}\right)$, nitrate (NO_3_^-^). Using pH/EC/TDS meter (Hanna HI 9811-5), the EC, pH and TDS of water samples were measured in the field immediately after the collection of the samples. Calcium, magnesium, chloride, carbonate and bicarbonate were analyzed by a titration method using the standard procedure as given in APHA (1995). TH was measured by a titration method using a standard hydrochloric acid solution and a standard EDTA solution. Sodium and potassium concentrations were determined using a flame photometer (Systronics, 130). Sulphate and nitrate were determined using a UV-visible spectrophotometer (Spectronic, 21, BAUSCH and LOMB). The fluoride concentration in water was determined electrochemically, using a fluoride ion-selective electrode (ISE) with an Orion 4 star meter benchtop pH/ISE meter [3]. Standard fluoride solutions (0.1–10 mg/L) were prepared from a stock solution (100 mg/L) of sodium fluoride. The ion meter was calibrated for a slope of −59.2±2 [3]. As per experimental requirements, a 2 mL aliquot of total ionic strength adjusting buffer grade III (TISAB III) was added in 20 mL of each groundwater sample before the fluoride concentration was determined.
